# CREB3L1 and PTN expressions correlate with prognosis of brain glioma patients

**DOI:** 10.1042/BSR20170100

**Published:** 2018-05-22

**Authors:** Li-qiang Liu, Li-fei Feng, Cheng-rui Nan, Zong-mao Zhao

**Affiliations:** 1 Neurosurgical Department, The Second Hospital of Hebei Medical University, Shijiazhuang, Hebei 050000, China; 2 Neurosurgical Department, Xingtai People’s Hospital, Xingtai, Hebei 054001, China

**Keywords:** CREB3L1, brain gliomas, PTN, survival time and prognosis of gliomas

## Abstract

The present study was conducted to investigate the clinical significance of cAMP responsive element binding protein 3 like 1 (CREB3L1) and pleiotrophin (PTN) expression in prognosis of patients with brain gliomas. Human brain tissue samples were collected from normal glial tissues (control), low- and high-grade glioma tissues. CREB3L1 and PTN expression levels in cells were assessed by immunohistochemistry (IHC), and population distribution of the CREB3L1- and PTN-presenting patients was examined. The *CREB3L1* and *PTN* mRNA expression levels in three types of the brain cells was determined by RT-PCR. Survival rates for population of the CREB3L1- and PTN-presenting patients were examined. CREB3L1^+^ cell counts were decreased with increased PTN^+^ cells in the low-grade and high-grade glioma tissues as compared with the control. Population proportion of the CREB3L1^+^-presenting patients decreased from the control to the high-grade glioma and the population of the PTN^+^-presenting patients increased in low- and high-grade gliomas as compared with the control (both *P*<0.05). The decrease in the *CREB3L1* mRNA expression was associated with the increase in the *PTN* mRNA expression in the low- and high-grade gliomas (*P*<0.05). Survival time for patients with CREB3L1^−^ and PTN^+^ gliomas was shorter than patients with CREB3L1^+^ and PTN^−^ gliomas in the investigated cohorts (both *P*<0.05). There was a relationship between the expression levels of both proteins and survival time. CREB3L1 and PTN expression levels serve as biomarkers with utility in grading gliomas. Absence of CREB3L1 and presence of PTN in brain glioma cells correlate with survival time of the glioma patients.

## Introduction

Most tumors in the brain are gliomas originating in the glial cells and are often diagnosed as malignant brain tumors [1]. Gliomas can be life-threatening depending on the grade of malignancy. Grades III and IV are the highest grades based on tumor’s growth potential and aggressiveness, with the worst prognosis and greatest need for the most aggressive treatment [2–4]. The exact cause of glioma is not known and therefore patients have a median life expectancy following diagnosis and aggressive treatment of just 14 months [5].

cAMP responsive element binding protein 3 like 1 (CREB3L1), a member of the unfolded protein response, has recently been identified as a metastasis suppressor in both breast and bladder cancer [6]. CREB3L1 expression is frequently altered in many cancer types suggesting that it could have a broader role in cancer progression and metastasis [7,8]. Pleiotrophin (PTN) is an angiogenic and mitogenic growth factor for various cell types and produced by some human tumors [9,10]. The tumor-secreted PTN is supposed to contribute to tumor’s malignancy by targetting endothelial and microglial cells [11].

The purpose of the present study was to explore clinical roles of CREB3L1 and PTN expression in brain glioma progression. Our results indicate that the CREB3L1 and PTN expression are useful indicators that help to identify the nature of the glial cells and malignant degrees of brain gliomas. Furthermore, changes in the CREB3L1 and PTN expression levels in the tumor cells offer the potential to assess prognosis of the glioma patients.

## Materials and methods

### Patient and inclusion criteria

We recruited 42 patients with glial brain tumors requiring surgical resection in the period from February 2014 to June 2014. These patients included 23 males and 19 females aged 5–72 (median: 44.8 years). Normal brain tissue samples were obtained from nine patients who underwent a partial excision of the brain tissue due to traumatic brain injury or intracerebral hemorrhage. There were no statistical differences in the distribution of age and gender between any two groups (both *P*>0.05).

Major inclusion criteria were as follows: (i) histopathological diagnosis as brain gliomas; (ii) glioma grade from I to IV by signs of cell division under the light microscope and categorized as low- (I–II) and high-grade (III–IV) gliomas according to WHO grading system [12]; (iii) no radiotherapy, chemotherapy, or immunotherapy prior to surgical resection of the brain tumors; (iv) all patients received a 30-month follow-up and survival survey after surgery.

The study has been approved by the Ethics Committee of the Second Affiliated Hospital of Hebei Medical University. The Ethics Committee also approved the related tissue collection from these patients based on the experimental design and analysis of clinical outcomes. All subjects signed written informed consent forms for the present study.

### Preparation of brain specimens

Brain tissue samples were collected from patients with traumatic brain injury (control) or glial brain tumors after surgical excision. Each sample was placed in a wide-mouth bottle filled up in 4% (v/v) formalin for the immunohistochemistry (IHC) examination. Some samples prepared for RT-PCR (qPCR) were immediately snap-frozen in liquid nitrogen and then stored at −70°C until use.

For immunohistochemical detection, cross-sections of glial brain tissues were embedded into paraffin and then sliced on a microtome at 4 μm. The slices were mounted on slides, dehydrated using alcohol washes of increasing concentrations (75, 80, 90, 95, and 100%) for different time periods, and cleared using a detergent like xylene before being imaged under a microscope. A two-step indirect IHC staining method was employed in the present study. Briefly, the primary rabbit antibodies (Abam, U.S.A.) directed against human CREB3L1 and PTN were diluted to 1:200 and 1:250, respectively. The slides from individual samples were stained with the antibodies at 4°C overnight. A horseradish peroxidase-conjugated secondary antibody (goat anti-rabbit, Beijing ComWin Biotech Co., Ltd) reacting with 3,3′-Diaminobenzidine (DAB) substrate-chromogen was intended for use on the formalin-fixed, paraffin-embedded tissue sections. Reaction with DAB on the sections can produce a brown product at the site of the target antigen that is insoluble in alcohol and xylene. Slides were washed three times with PBS before being analyzed using a light microscope (Olympus, Japan) at a 200-fold magnification for visualizing expression of the targets in the investigated tissues during the disease process.

### Application of IHC

Five circular areas from 200 high-power fields on each slide were randomly selected and viewed by a pathologist under light microscope. The relative proportions of the glial cells and glioma cells from a total of 200 cells/slide were determined in each specimen based on the number of the stained cells and their scores according to immunoreactive Remmele scores (IRS) [13]. Positive responses for the CREB3L1 and PTN staining were indicated by yellow brown colors seen in the staining cells.

In the present study, immunostaining of the glial brain cells was observed under a light microscope. According to IRS, a standard for negative (−) or a positive (+) effect of CREB3L1- and PTN-staining cells is defined as cells of ≤4 or >4% in the total cell counts in each microscopic area, respectively. Population proportion for CREB3L1^+^ and PTN^+^ patients was calculated according to the number of the patients with the positive cell staining in their brain tissue specimens.

### Determination of *CREB3L1* and *PTN* mRNA levels

The target-specific qPCR primers were designed and chosen using Primer-BLAST (NCBI/Primer-BLAST) [14]. *CREB3L1* and *PTN* mRNA expression levels were assessed by using SYBR Premix Ex Taq™ (Fulengen Co., Ltd., Guangzhou, P.R. China) and mRNA levels were normalized to *GAPDH* housekeeping gene. The following sense and antisense primer sequences were used: CREB3L1, 5′-CCACGAGACCACCAAGTACC-3′ and 5′-GTACCAGGGGTCCGTCCTAT-3′; PTN, 5′-AGAAGCAATTTGGCGCGGA-3′ and 5′-TGCACCACCAACTGCTTAGC-3′; GAPDH, 5′-GGCATGGACTGTGGTCATGAG-3′ and 5′-TGCACCACCAACTGCTTAGC-3′. Briefly, the total RNA from glial and glioma tissues in brain was extracted using a tissue homogenizer in lysis buffer and purification of RNA was performed with RNeasy minicolumns following the manufacturer’s protocol (Fulengen Co., Ltd, Guangzhou). RNA was quantitated using the NanoDrop ND-1000 spectrophotometer and amplified and biotin-labeled with Nugen’s Ovation System. The yield of total RNA per replicate varied from 0.6 to 2.0 μg. Fifty nanograms of RNA was added in a SYBR qPCR Master Mix for real-time qPCR. Quantitative data of *CREB3L1* and *PTN* mRNA after normalizing to GAPDH were shown with a fold change compared with the expression level of the control sample.

### Survival function of patients with gliomas

The Kaplan–Meier estimate is the simplest way of computing the survival over time in spite of all the difficulties associated with subjects or situations [15]. The survival curve can be created assuming the patient’s prognosis in a statistical graph showing the percentage surviving in the investigated cohort. Points on the curve indicate the proportion or percentage survival at a particular time (month) after the start of this observation.

### Statistical analyses

Data were expressed as a percentage of population proportion in the study cohort and a sample median of an interquartile range (IQR). Statistical analysis was performed using Statistical Package for the Social Science (SPSS, version 16.0). One-way ANOVA was implemented for comparison of independent variables. Student’s paired *t* test was used to compare measurements of cells stained by antibodies. The Chi-square test (χ^2^) was conducted to analyze the significance of population distribution for the CREB3L1- and PTN-presenting patients. A *P*-value of <0.05 was considered significant.

## Results

### Identification of glial brain cells

**Figure 1:**
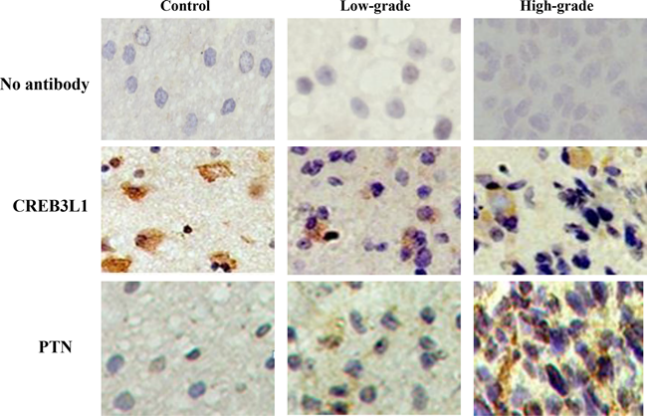
Microscopic veiw of brain glioma cells Glial cells from brain specimens were observed in the absence (upper) and presence of antibodies to CREB3L1 (middle) and PTN (down). The CREB3L1- and PTN-positive cells were shown in a brown color under a light microscope (×200), respectively. There were statistical differences in the CREB3L1 and PTN staining cell counts between any two groups (all *P*<0.05, *n*=5).

Glial cells from brain specimens were observed in absence and presence of specific antibodies to CREB3L1 and PTN. The results are shown in Figure 1. Types of brain glioma were categorized by apprearance as oligodendrogliomas according to the features of oligodendrocytes having a fried-egg shape or short arms in appearance of the cells. These cells did not have staining when they were only incubated with the secondary antibody. The CREB3L1- and PTN-positive cells presented in a brown color under a light microscope (×200). Five circular areas in each slide were examined. CREB3L1^+^ cell counts were reduced from the normal as compared with the high-grade glioma tissues. The ranking for the diminished counts were shown in such an order of high-grade > low-grade glioma > the control in the investigated tissues. There were a statistically significant differences in the counts between any two groups (*P*<0.05, *n*=5).

PTN^+^ cell counts significantly increased in low- and high-grade gliomas as compared with the control. The increased counts were displayed in such an order of high grade > low grade > the control in these samples. There were statistical differences in the counts between any two groups (*P*<0.05, *n*=5).

### Distribution of CREB3L1- and PTN-expressed population

**Figure 2:**
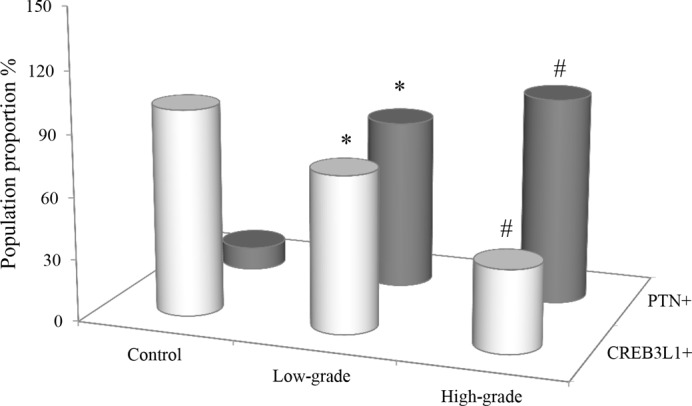
Comparison of population proportions for the CREB3L1- and PTN-presenting patients Population distribution for the CREB3L1- and PTN-presenting patients were expressed as a percentage of the control (*n*=9) and the cohorts with the low- (*n*=17) and high-grade (*n*=25) gliomas. χ^2^ test was used to observe the changes of the proportions with CREB3L1^+^ and PTN^+^. *: *P*-value of <0.05 compared with the control; ^#^: *P*-value of <0.05 compared with either the control or the low-grade glioma.

The distribution of CREB3L1- and PTN-staining patients was examined in the study cohort and the results are shown in Figure 2 and Table 1. All of the control subjects were CREB3L1^+^ (*n*=9). In contrast with the control, the population proportion for the CREB3L1^+^ staining was reduced to 76 and 40% in the patients with low- and high-grade gliomas (*n*=17 and 25), respectively. There were statistical differences in the reduced proportions between any two groups of the CREB3L1-presenting patients (χ^2^ test, *P*<0.05).

**Table 1:** Distribution of CREB3L1- and PTN-positive people

	Number (%)	
	CREB3L1+	PTN+
Control (*n*=9)	9 (100)	1 (11)
Low-grade (*n*=17)	13 (76)*	14 (82)*
High-grade (*n*=25)	10 (40)†	25 (100)†

* *P*<0.05 compared with the control; ^†^*P*<0.05 compared with either the control or the low-grade glioma.

The percentage of PTN^+^ patients was significantly increased in high- and low-grade gliomas (*n*=17 and 25) as compared with a positive population in the control (*n*=9), respectively. The numbers of the patients with low- and high-grade tumors reached 82 and 100% increments as compared with the control (11%). There were statistical differences in the distributions of the positive patients between any two groups (χ^2^ test, *P*<0.05).

### *CREB3L1* and *PTN* mRNA expression in glioma cells

**Figure 3:**
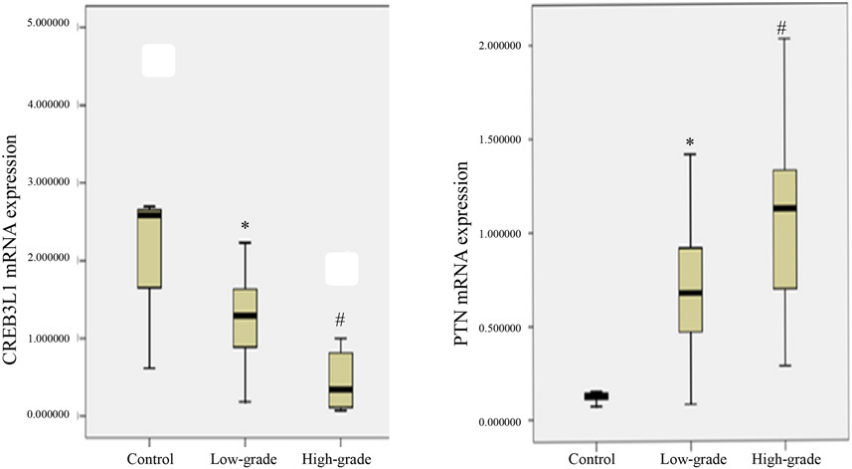
*CREB3L1* and *PTN* mRNA expression levels *CREB3L1* (left) and *PTN* (right) mRNA expression was examined in control, low- and high-grade glioma cells (*n*=5 in each group). The data were expressed as a fold change in the mRNA expression level of the individual tissues. The *CREB3L1* and *PTN* mRNA levels were calculated in reference to the control mRNA level. The results were shown as median (IQR). *: *P*-value of <0.05 compared with the control. ^#^: *P*-value of <0.05 compared with either the control or the low-grade glioma.

*CREB3L1* and *PTN* mRNA expression was measured in the brain specimens obtained from the control glial cells, low- and high-grade glioma cells. The *CREB3L1* and *PTN* mRNA expression levels in brain tissue samples were expressed as median (IQR) and the results are shown in Figure 3. A relative level for the *CREB3L1* mRNA expression was displayed as 2.59 (1.48) in the control cells, 1.30 (0.89) and 0.34 (0.75) in the low- and high-grade glioma cells, respectively. The *CREB3L1* mRNA expression levels in the low- and high-grade glioma cells were 1.99- and 7.59-fold lower than the control. There were statistical differences in the mRNA expression levels between any groups (*P*<0.05).

In terms of PTN examination, a median value (IQR) for the *PTN* mRNA expression levels were shown as 0.13 (0.04) in the control, 0.67 (0.58) and 1.12 (0.66) in the low- and high-grade gliomas, respectively. The *PTN* mRNA expressions levels in low- and high-grade gliomas were obviously changed considerably with 5.15- and 8.62-fold increases over the control. In contrast, there were statistical differences in the expression levels between any two groups (*P*<0.05, *n*=5).

### Prognosis of CREB3L1- and PTN-presenting patients

**Figure 4:**
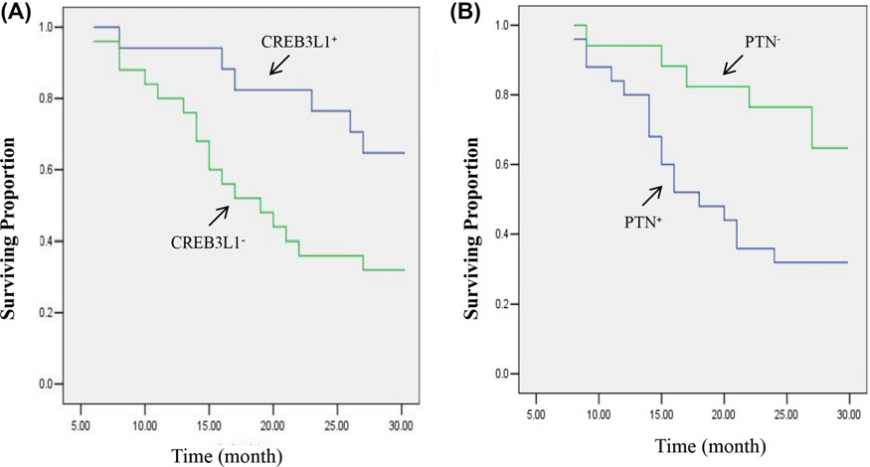
Observation of survival curves for CREB3L1- and PTN-presenting patients Survival curves of patients were plotted over time (months) on the graph. Population proportion for the survivors with CREB3L1^−^ (left) and PTN^+^ (right) significantly decreased as compared with the survivors with CREB3L1^+^ and PTN^−^. Data were calculated with a percentage of the survival proportion in each group. Statistical test showed a *P*-value of <0.05 in the numbers of survivors between the two cohorts.

Comparative survival curves for the patients with glial brain tumors were examined according to CREB3L1 and PTN expression levels. The curves displayed survival in the study cohort plotted over time. The results are shown in Figure 4. The survival curve for the patients with CREB3L1^+^ stayed in high plateau as compared with the patients with CREB3L1^−^. These two survival curves for the CREB3L1-expressing patients descended at different rates during the time period of 30 months. In contrast with the CREB3L1-positive patients, a decrease in survival proportion (%) for the CREB3L1-negative patients was more obvious with an initial decrement observed at the time point of the seventh month after the brain glioma was surgically excised (Figure 4A). With expanding time, the number of the survivors in the cohort reached ~35% with a 30% decrease as compared with the patients with CREB3L1^+^ at the time point of 30 h. There was a positive correlation between its expression level and survival rate (r = 0.387, *P*=0.046).

The survival curves for the PTN-expressing patients also descended at different rates during the time period of 30 months (Figure 4B). The curve for the patients with PTN^−^ was presented at the top of the graph. In further analysis, surviving proportion for these patients arrived to 65% or more at the 30th month. In terms of PTN^+^ patients, survival curve started to drop down at the 7th month. Surviving proportion for those patients continuously moved down with the prolonging time course and reached 32% in the survival population at the end of the experiment. There was a 33% margin in the difference of survival rate between the patients with PTN^+^ and PTN^−^. A negative correlation was indicated between the two variables of the PTN expression level and the survival rate (r = −0.731, *P*=0.021).

## Discussion

In the present study, the numbers of CREB3L1^+^ cells in the same microscope’s field of view gradually decreased from the control to the high-grade glioma, whereas the PTN^+^ cell counts increased in the low- and high-grade glioma tissues as compared with the control. These findings led us to speculate that the changes in the CREB3L1 and PTN expression not only benefit to estimate the nature of the glial cells but also offered clinical clues to help realize the degree of malignancy of the brain glioma tumors. It has been reported that loss of CREB3L1 expression may contribute to or be required for cancer progression and the development of a metastatic phenotype [8,16,17]. Conversely, PTN expression is increased in a number of human cancers, in particular brain tumors [11,18] and is thought to be involved in tumor angiogenesis [19]. Based on our data and these reports, it is conceivable that the changes in the CREB3L1 and PTN expression levels were related to the aggressive behaviors (invasion and metastasis) of the glioma cells in disease progression.

Population distribution for the CREB3L1- and PTN-presenting patients was examined in patients with low- and high-grade gliomas, as well as a control group. In contrast with the control all with CREB3L1^+^, a proportion for the CREB3L1-presenting population gradually reduced to 76 and 40% in the survivors with the low- and high-grade gliomas. Concurrently, population proportion for the PTN^+^ cells progressively increased to 82 and 100% in the cohorts with the low- and high-grade gliomas as compared with the control of 11%. These results were completely consistent with the observations of the tumor cells at the protein level, suggesting that the changes of the two protein expressions were valuable indicators in assessing glioma progression and the disease severity. CREB3L1 and PTN expressions have been involved in human gliomas [11,20]. Furthermore, activation and/or inhibition of these protein expressions on cancer cells have shown the potential interest for cancer therapy [17,21]. Given that the PTN protein was overexpressed in the brain glioma patients, its blockade could be a potential target for a treatment strategy of the gliomas as the tumors still remain a therapeutic challenge [22].

The *CREB3L1* mRNA level in the brain specimens displayed a gradient decrease with an increase in the *PTN* mRNA level from the control to high-grade glioma tissues. These findings showed statistical differences in the *CREB3L1* and *PTN* mRNA levels between any two types of the collected tissues, suggesting an important molecular mechanism contributing to progression of glial tumors with the growth potential. Since these changes of the glioma tumors at the gene level were identical with the results observed at their protein levels, it is reasonable to consider that CREB3L1 and PTN serve as biomarkers for helping to identify the nature of the glial cells and evaluating malignant degrees of brain gliomas. Down-regulation of *CREB3L1* gene has been found in two cancer types [6,8]. Insufficiency of the gene expression induces proliferation of cancer cells and facilitates tumor cell spreading and migration [8,16]. Though the mechanism(s) for lack of *CREB3L1* gene expression is not clear in tumor progression, methylation of the gene could be a cause involved in negatively regulating *CREB3L1* mRNA expression in breast cancer cells [6,23]. *PTN* mRNA was detected in high-grade gliomas since the tumors constitutively express and secrete PTN [11]. Up-regulation of PTM gene expression has been found based on the reports in which the human *PTN* gene localized on chromosome 7 is often amplified in gliomas [24,25]. Since lack of CREB3L1 expression results in increasing expression of PTN to positively regulate angiogenesis and facilitate the growth of tumors [7], it is very likely that a combining measure of the *CREB3L1* and *PTN* gene levels in gliomas would be advantageous to a single examination in understanding malignant features of the tumors in clinical practice.

To explore a clinical significance of the CREB3L1 and PTN expression levels, survival rates for the CREB3L1- and PTN-presenting patients were analyzed over a time period of 30 months.

Due to all the patients scoring CREB3L1^+^ with degrees of PTN expression in the study cohort, the survival rates for the patients were compared based on the separate changes in the CREB3L1 and PTN expression levels. Our results revealed that percentages of survivors with CREB3L1^−^ and/or PNT^+^ started to drop at the seventh month. With expanding time, the number of the survivors in the patients with CREB3L1^−^ and PTN^+^ reached ~35 and 32% with 30 and 33% decreases as compared with the CREB3L1^+^ and PTN^−^ survivors at the time point of 30 h, respectively. These findings indicated that absence of CREB3L1 and presence of PTN expression in the tumor cells obviously shortened the length of survival time. In statistical analysis, there was negative or positive correlation between the changes in CREB3L1 and PTN expression and the numbers of the survivors. The results supported the consideration that a combining measure of both protein expressions was more conclusive than a single measure of the proteins in assessing development of brain gliomas. Since the decrease in the survivors with CREB3L1^−^ and PTN^+^ reflected the changes in malignant degree of the tumors, it is conceivable that the states of CREB3L1 and PTN expression are regarded to be a diagnostic tool serving for predicting a poor prognosis of the brain glioma and evaluating the efficacy of newly targetted molecular drugs. Malignant gliomas are diffuse, highly invasive, and often multifocal tumors that have a dismal prognosis with several months [26,27]. One of the major obstacles to effective treatment of gliomas is the intrinsic infiltration ability of single tumor cell that depends on the grade degree of gliomas, resulting in incomplete surgical removal and the high frequency of tumor recurrence [28]. Our study could provide an important clue to the clinical roles of CREB3L1 and PTN expressions in evaluating grade-versions of brain gliomas, and thus may offer an appropriately therapeutic option for the cancer patients.

In conclusion, the present study indicates that the CREB3L1 and PTN expressions provide a clinical cue in helping to realize grade of brain glioma cells. Absence of CREB3L1 and presence of PTN expression in the tumor cells correlate with a poor prognosis of the glioma patients.

## Abbreviations

CREB3L1: cAMP responsive element binding protein 3 like 1
DAB: 3,3′-diaminobenzidine
IHC: immunohistochemistry
IQR: interquartile range
IRS: immunoreactive Remmele score
PTN: pleiotrophin
